# Multiple Primary Lung Cancers: Treatment Outcomes After Stereotactic Body Radiotherapy (SBRT)

**DOI:** 10.7759/cureus.41319

**Published:** 2023-07-03

**Authors:** Gyujae Choi, George Chen, Weiyuan Mai, Albert Chen, Angela Zhu

**Affiliations:** 1 Radiation Oncology, Baylor College of Medicine, Houston, USA; 2 Radiation Oncology, Multicare Health System, Tacoma, USA; 3 Radiation Oncology, Michael E. Debakey Veterans Affairs Medical Center, Houston, USA; 4 Radiation Oncology, Michael E. DeBakey Veterans Affairs Medical Center, Houston, USA

**Keywords:** metachronous lung tumors, synchronous lung tumors, multiple primary cancer, stereotactic ablative body radiotherapy, non-small cell lung carcinoma (nsclc)

## Abstract

Purpose/objectives: Patients with lung cancer sometimes present with multiple primary lung cancers (MPLCs), either simultaneously (synchronous) or after treatment of an initial lesion (metachronous). Although open surgery remains a treatment mainstay for patients with stage I-II non-small-cell lung cancer (NSCLC), stereotactic body radiation therapy (SBRT) is an acceptable alternative for patients who are medically unfit for or who refuse surgery. In this study, we retrospectively examine the outcome among patients with early-stage MPLCs treated at our institution with SBRT.

Materials/methods: Patients at our institution receiving SBRT for MPLC between June 2011 and March 2020 were reviewed retrospectively. Prior to undergoing definitive SBRT, the imaging, and pathology for every patient were reviewed in a multi-disciplinary thoracic/pulmonary tumor board. Dose and fractionation varied with the most common prescriptions being 50 Gy/5 fractions, 56 Gy/4 fractions, and 55 Gy/5 fractions.

Results: A total of 38 patients with a total of 80 MPLCs were treated, among which 68 were T1 lesions and 12 were T2 lesions. Median follow-up was 25.9 months, with local control (LC) rates calculated per lesion to be 98.6%, 93.3%, and 88.2% at one, two, and three years. Median overall survival (OS) was 43.5 months; 83.6%, 67.8%, and 52.3% at one, two, and three years, respectively. Sixty-two of the 80 (77.5%) treated lesions were not associated with any subsequent acute or late toxicity. The 18 (22.5%) lesions associated with toxicity included nine acute and nine late events. All toxicity was either grade 1 (13 of 18) or grade 2 (five of 18).

Conclusions: SBRT for early-stage MPLC achieves high control rates with limited toxicity. MPLC patients deemed unfit for open surgical management should be considered for definitive SBRT.

## Introduction

Patients with lung cancer sometimes present with multiple primary lung cancers (MPLCs), whether diagnosed with two simultaneous primary cancers (synchronous) or developing a second lesion after treatment of their initial disease (metachronous). The incidence of MPLCs has been reported to range from 3.7% to 8%, a finding linked to tobacco carcinogens damaging the entire field at risk [1].

Proper staging of an MPLC can significantly impact patient treatment options and prognosis [2]. If the two lesions are considered separate disease processes, then the patient has each lesion staged separately according to the Tumor, Node, Metastasis (TNM), eighth edition staging guidelines by the American Joint Committee on Cancer (AJCC). If a second lesion is considered a satellite nodule of the index lesion, the patient’s disease is then classified as T3 (nodule in same lung lobe), T4 (nodule in separate lobe in the ipsilateral lung), or M1a (nodule in the contralateral lung). Misclassification of a patient with contralateral lung nodules to M1a staging may lead to inappropriate use of palliative treatment options.

Surgical resection dominates the treatment of stage I-II non-small-cell lung cancer (NSCLC), while stereotactic body radiation therapy (SBRT) is deemed an acceptable alternative for patients that are medically unfit or who refuse surgery [3]. Previous retrospective studies have reported the outcome of MPLC treated with SBRT alone, or in combination with other methods [4-9]. However, such studies were small in scale and included a limited number of cases. Considering this shortcoming, we investigated our institutional experience in treating MPLC patients.

This article was previously presented as a meeting abstract at the 2023 Radiosurgery Society Scientific Meeting on March 23, 2023.

## Materials and methods

This retrospective study was approved by Baylor College of Medicine's Institutional Review Board (H-46948) and Veteran Affair Research and Development Committee (ID# 21505 H), and the need for informed consent was waived. Between June 2011 and March 2020, the medical records for 328 patients receiving definitive SBRT for lung nodules at the Michael E. DeBakey VA Medical Center were identified via treatment records and reviewed by Choi and Zhu. At presentation, all such patients had previously undergone a review of imaging (CT and/or FDG-PET imaging) and pathology during a multi-disciplinary thoracic/pulmonary tumor board. A multi-factorial analysis, with or without histological confirmation, was originally used to arrive at a diagnosis of MPLC. Patients were included in final analyses if they received SBRT exclusively for their first two or more lesions. Patients who underwent prior surgical resection or lung radiotherapy were excluded, as were those with nodal disease or extrathoracic metastases.

The SBRT method used for this study began with three-dimensional (3D) chest computerized tomography (CT) performed at maximum end-expiration. Treatment plans were designed using Accuray’s Precision treatment planning software (Sunnyvale, CA) and Monte Carlo calculation algorithms. Plans were optimized to limit the dose to surrounding organs at risk (OARs), which included the heart, esophagus, bilateral lungs, great vessels, spinal cord, trachea, and proximal bronchial tree. Fiducial tracking with synchrony was used during all treatment delivery. During this retrospective study, dose and fractionation varied; however, the most common prescriptions were 50 Gy/5 fractions, 56 Gy/4 fractions, and 55 Gy/5 fractions, delivered to the planning target volume (PTV) to the 95%-100% isodose lines.

Post SBRT, all patients underwent regular clinical and imaging (CT) follow-up at three-, six-, and 12-month intervals, with FDG-PET imaging generally reserved for suspected recurrent disease. Kaplan-Meier methodology was used to calculate local control (LC) and overall survival (OS) as measured from the completion of the patients’ second course of SBRT (or third if patients received three courses of SBRT), while a log-rank test was utilized to compare variables. LC was calculated on a per-lesion basis and defined as the in-field progression of disease within the PTV. Relationships between demographic and treatment variables and OS or LC were analyzed using the Cox proportional hazards model and analysis of covariance (ANCOVA). All p-values report represent two-sided statistical tests; the predetermined value for significant was p = 0.05. All statistical analyses were performed using STATA/IC (version 16.1, College Station, TX). Toxicity was determined using Common Terminology Criteria for Adverse Events (CTCAE), v5.

## Results

Patient and tumor characteristics

A total of 38 patients fulfilled our inclusion criteria and received multiple courses of lung SBRT for a total of 80 lesions. The median age of the cohort was 70.5 (range 61.2-92.0) with a median Karnofsky Performance Scale (KPS) of 80% (range 60%-100%). All patients who met inclusion criteria were male and had pathologic confirmation of their primary lesion; 33 out of 38 (87%) had pathology documented for their second lesion. Twenty-two of 38 (58%) patients presented with synchronous lesions, while the other 16 (42%) patients presented with metachronous lesions. Twenty-one of the 22 patients with synchronous presentations underwent nodal staging via endobronchial ultrasound (EBUS) and biopsy. Of the 16 patients with metachronous presentation, 11 patients underwent EBUS for their first lesion with five of these also undergoing EBUS for their second lesion. One of the five patients who had not undergone EBUS for their first lesion did so for their second lesion. Thirty-six of the 38 patients were considered surgically or medically inoperable, while two patients refused surgery.

Twenty-two of 38 patients presented with bilateral disease (stage M1a), while 16 of 38 had a second lesion in the ipsilateral lung (T3 or T4). Of the latter, seven of 16 patients had a second lesion in the same lobe (T3). Four of the 38 patients had a third lesion presumed to be a primary, which was also treated with SBRT; of these, three patients had all three lesions in the same lobe, and the fourth patient had three lesions in the ipsilateral lung. The median tumor volume was 4.70 cc (range 0.29-49.79 cc). By TNM (eighth edition) size criteria, 68 of 80 lesions were stage T1 while 12 lesions were stage T2. Patient and tumor characteristics are summarized in Tables 1, 2, respectively. The median follow-up for the entire cohort was 25.9 months.

**Table 1:** Patient characteristics

Characteristic	Number of patients (%)
Caucasian	26/38 (68.4)
African-American	12/38 (31.6)
Synchronous presentation	22/38 (57.9)
Metachronous presentation	16/38 (42.1)
Ipsilateral presentation	16/38 (42.1)
Bilateral presentation	22/38 (57.9)
Same lobe presentation	7/38 (18.4)
Different lobe presentation	31/38 (81.6)

**Table 2:** Tumor characteristics

Characteristic	Number of tumors (%)
Squamous cell carcinoma (SCC)	32/80 (40.0)
Adenocarcinoma (AC)	38/80 (47.5)
Other histology or no biopsy	10/80 (12.5)
Tumor stage: T1	68/80 (85.0)
Tumor stage: T2	12/80 (15.0)

**Figure 1:**
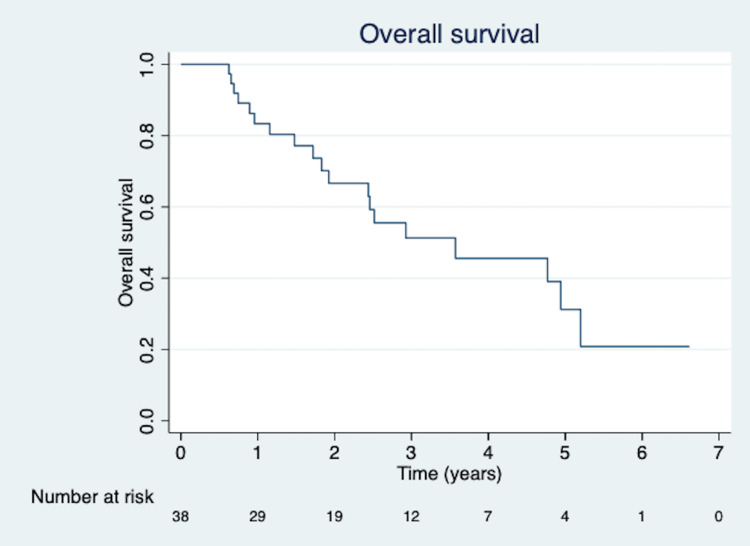
Kaplan-Meier curve for overall survival

**Figure 2:**
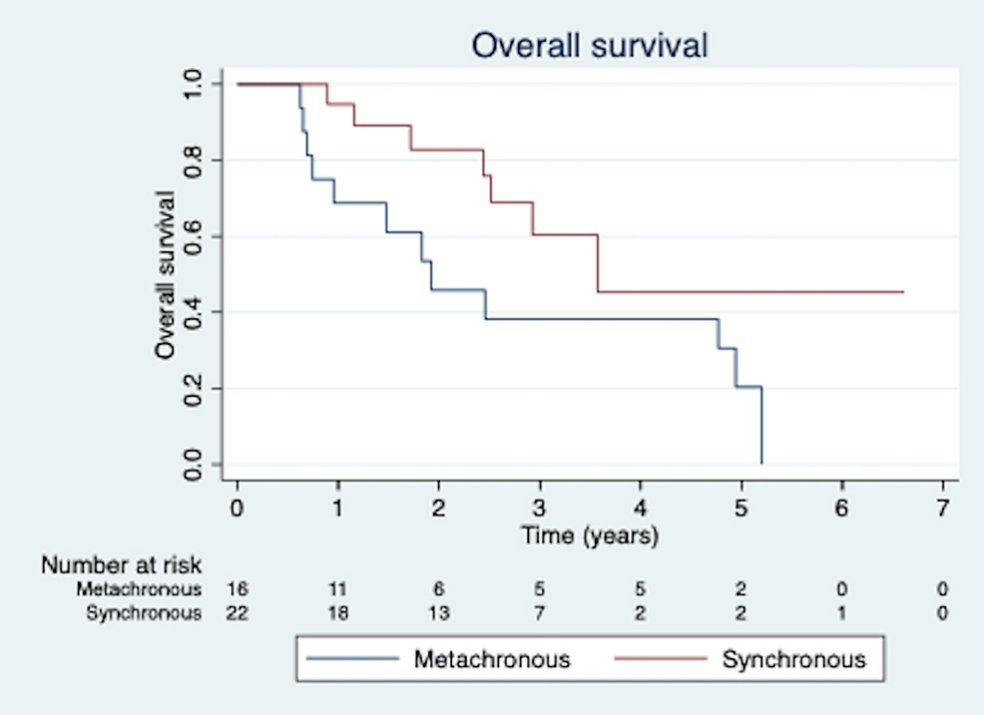
Kaplan-Meier curve for overall survival based on synchronous presentation

Median OS was 43.5 months from the completion of the patients’ second (or third if relevant) course of SBRT (6.3 to 80.5 months). OS rates were 83.6%, 67.8%, and 52.3% at one, two, and three years (Figure 1). Age was a significant predictor of decreased OS on multivariate analysis (p = 0.002). Metachronous tumor presentation predicted decreased OS on univariate analysis (p = 0.049) but not multivariate analysis (p = 0.146; Figure 2). There was no association between OS and same-lobe or ipsilateral lung presentation of the second primary lesion, biopsy confirmation of the second lesion, nodal staging status for both lesions, smoking pack years, and KPS.

**Figure 3:**
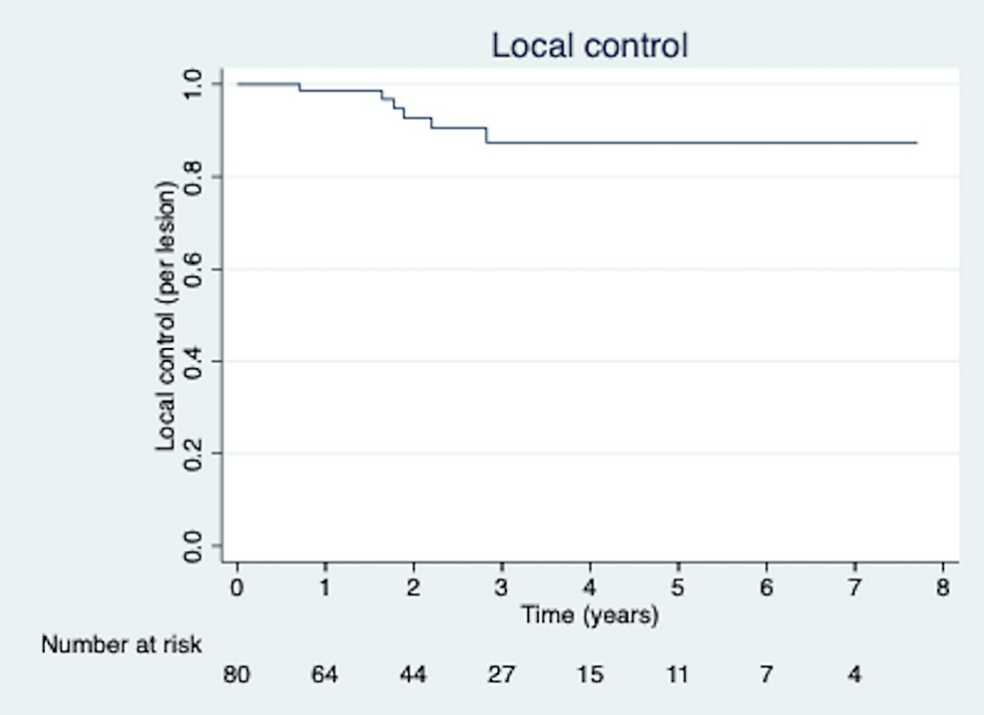
Kaplan-Meier curve for local control

Among the 80 total lesions treated with SBRT in our cohort, there was a total of six recurrences. Rates of LC, calculated on a per-lesion basis, were 98.6%, 93.3%, and 88.2% at one, two, and three years, respectively (Figure 3). Gross tumor volume (GTV) was a significant predictor of decreased LC on multivariate analysis (p = 0.01). Tumor histology and biologically effective dose (BED) of delivered radiotherapy were not significant predictors of LC.

Sixty-two of the 80 (77.5%) treated lesions were not associated with any subsequent acute or late toxicity. Treatment toxicity was observed in 18 (22.5%) lesions and included nine acute and nine late events. All observed toxicity was grade 1 (13 of 18) or grade 2 (five of 18). A summary of patient-reported toxicity can be found in Table 3.

**Table 3:** Patient-reported acute/late toxicity

Toxicity		
Acute: chest wall pain	G1: 6 events	G2: 2 events
Acute: rash	G1: 1 event	G2: 0 events
Late: chest wall pain	G1: 5 events	G2: 2 events
Late: pleuritic pain	G1: 1 event	G2: 1 event

## Discussion

Although surgery is widely accepted as the standard of care for operable, early-stage NSCLC, to date there are no randomized data in head-to-head studies that demonstrate the non-inferiority of radiotherapy relative to surgery. The ongoing VALOR trial (NCT02984761) comparing definitive surgery and SBRT will attempt to clarify this treatment paradigm. However, for those unfit for surgery or who refuse surgery, SBRT is generally considered an acceptable alternative to surgical resection.

There exists limited data in radiotherapy literature on SBRT treatment of MPLCs, and this study represents one of the largest reported institutional experiences of lung SBRT for MPLCs. Of note, the present cohort is focused on non-metastatic patients who had not previously been treated at all - in essence, a “pure” analysis of SBRT for MPLCs.

Chang et al. [6] reported on 101 patients with 130 synchronous and metachronous MPLCs treated with SBRT for their second lesion, 29 of whom received SBRT to both lesions. Chang et al. observed excellent two-year results for LC (97.4%) and OS (73.2%). Creach et al. [7] published their experience using SBRT to treat 63 patients harboring a total of 76 total synchronous and metachronous MPLCs, observing a median progression-free survival (PFS) and OS of 15.5 and 20 months, respectively. Shintani et al. [8] described median PFS and OS of 25.3 and 45.6 months, respectively, within a cohort of 18 patients presenting with synchronous lesions. The experience of Griffioen et al. [9] likely represents the closest comparison to our study’s patient cohort. Their study included 62 patients treated with SBRT for synchronous MPLCs, 56 of whom received SBRT to both lesions, with a median OS of 31 months and two- and three-year LC rates of 84% and 78%, respectively.

Our current study’s survival and control rates compare favorably with the above previous reports. In the present report, rates of LC, as calculated per lesion, are on par with previous studies [10,11] also examining outcomes of SBRT in treating early-stage NSCLC. While the OS rate we found is more comparable to those of inoperable (as opposed to operable) early-stage NSCLC patients treated with SBRT [12,13], this is not surprising given the medical comorbidities of the present cohort.

A previous study found an association between synchronous disease presentation and worse PFS and OS outcomes, theorizing that these patients were presenting with early metastatic disease [7]. This was not the case in our study - there was no association between the chronology of presentation and OS on multivariate analysis. Patients with synchronous presentation demonstrated superior OS on univariate analysis, perhaps suggesting that these patients presented with true MPLCs while the metachronous cohort in fact included patients with early metastatic disease. This is bolstered by the fact that 21 of 22 of patients with synchronous disease underwent nodal staging via EBUS, while nodal staging numbers for the metachronous cohort were not as impressive.

The main limitations of our study are its retrospective nature, a small number of patients, all-male nature, and lack of pathological determination of a second primary tumor versus a metastasis in some patients. Survival outcomes have previously been reported to be similar for patients treated with SBRT for early-stage NSCLC with or without pathologic confirmation. In our analysis, there was no difference in OS between patients who had pathologic confirmation of their second lesion and those who did not.

## Conclusions

This study represents one of the largest reported institutional experiences of lung SBRT for treatment-naïve, non-metastatic MPLCs. SBRT treatment for early-stage MPLC can achieve both high rates of tumor control and acceptable survival rates. It accomplishes these key goals with limited toxicity, notably no grade $\geq$ 3 toxicity. All patients deemed unfit for surgical management of MPLC should be considered for definitive SBRT.
